# Scholarly Opportunities for Medical Students and Residents in Canadian Medical Professional Organizations

**DOI:** 10.1177/23821205231164029

**Published:** 2023-03-20

**Authors:** Mitchell D Thatcher, Adam M Wandzura, Mckinley C Smith

**Affiliations:** 1 12371 University of Saskatchewan, College of Medicine, Saskatoon, Canada

**Keywords:** scholarly opportunities, medical specialty organizations, medical trainees

## Abstract

**Objectives:**

Participation in medical specialty organizations can provide medical students and residents with additional research, advocacy, networking, and leadership opportunities. Although past research has looked at individual specialties in the United States, little is known about trainee involvement in Canadian organizations. Therefore, the aim of this study is to review the opportunities available for medical students and residents within Canadian medical specialty organizations.

**Methods:**

The websites of 71 Canadian medical specialty organizations were reviewed to assess levels of trainee participation.

**Results:**

Of the 71 organizations reviewed, 42 (59%) allow medical students and 67 (94%) allow residents to become members. Most organizations allow trainees to attend their annual conference (83% for students and 93% for residents), and the mean cost of attending the most recent virtual conference was $114 (range: $0–$475) for students and $142 (range: $0–$475) for residents. Twenty-two organizations (31%) have travel awards for students and 37 (52%) have awards for residents. Research grants are available in 41 (58%) of organizations for students and 56 (79%) for residents. Formal mentorship programs exist in 16 (23%) organizations for students and 25 (35%) for residents.

**Conclusion:**

To our knowledge, this study highlights for the first time the scholarly opportunities available to trainees within Canadian medical specialty organizations.

## Introduction

Participation in professional societies may enhance the education of medical students and residents through additional research, advocacy, networking, and leadership opportunities. Future physicians may be unaware of these opportunities available to them. Current studies have explored trainee involvement within American professional organizations of various medical specialties, including dermatology,^1^ orthopedic surgery,^2^ otolaryngology,^3^ ophthalmology,^4^ and radiology.^5^ However, the level of involvement of trainees in Canadian professional organizations to our knowledge has yet to be described in the literature. Therefore, the aim of this study is to review the opportunities available for medical students and residents within Canadian medical specialty organizations.

## Methods

Three investigators (M.T., A.W., M.S.) independently reviewed the websites and bylaws of the 71 Canadian national specialty societies as recognized by the Royal College of Physicians and Surgeons^6^ and the College of Family Physicians of Canada in 2021. Policies on trainee involvement within the organizations were recorded, including membership, leadership positions, annual meeting attendance, ability to present research, travel and research funding, and formal mentorship programs. Societies were contacted by email in attempt to verify the accuracy of the information. The University of Saskatchewan Biomedical Ethics Board determined this observational study to be exempt from review (exemption waiver number E166 April 19th, 2021).

## Results

Of the 71 Canadian medical specialty organizations reviewed, 42 (59%) offer membership to medical students (Table 1), with 22 (52%) being free, and 67 (94%) offer membership to residents (Table 2), with 35 (52%) being free. The mean (SD) membership fees are $23 ($28) for medical students, ranging from $0 to $90, and $31 ($41) for residents, ranging from $0 to $175. There are 27 (38%) organizations that allow medical students to serve on committees and 50 (70%) that allow residents. Formal mentorship programs exist in 16 (23%) organizations for students and 25 (35%) for residents.

**Table 1.** Survey of medical organizations for medical students.

Society	Membership	Membership Cost (CAD$). Per year	Serve on Committees	Attend Conferences	Conference Fee (CAD$)^a^	Present Research	Travel Award	Research Grant/Award	Mentorship
Association of Medical Microbiology and Infectious Disease Canada	Yes	50	Yes	Yes	100	Yes	Yes	Yes	Yes
Canadian Academy of Child and Adolescent Psychiatry	Yes	0	No	Yes	0	Yes	nd	Yes	No
Canadian Academy of Geriatric Psychiatry	Yes	0	No	Yes	85	Yes	No	No	Yes
Canadian Academy of Psychiatry and the Law	No	N/A	No	Yes	12	No	No	No	No
Canadian Academy of Sport and Exercise Medicine	Yes	25	nd	Yes	75	Yes	No	No	No
Canadian Anesthesiologists’ Society	Yes	90	nd	Yes	40	Yes	Yes	Yes	No
Canadian Association for the Study of Liver	Yes	0	Yes	Yes	0	Yes	Yes	Yes	No
Canadian Association of Child Neurology	No	N/A	N/A	Yes	nc	nd	No	No	No
Canadian Association of Emergency Physicians	Yes	90	Yes	Yes	200	Yes	No	Yes	Yes
Canadian Association of Gastroenterology	Yes	50	Yes	Yes	100	Yes	Yes	Yes	Yes
Canadian Association of General Surgeons	Yes	50	No	Yes	250	Yes	Yes	Yes	No
Canadian Association of Interventional Cardiology	No	N/A	N/A	nd	nd	nd	nd	nd	nd
Canadian Association of Interventional Radiology	Yes	0	Yes	Yes	0	No	No	No	No
Canadian Association of Medical Biochemists	Yes	15	nd	nd	nd	nd	nd	nd	nd
Canadian Association of Medical Oncologists	No	N/A	N/A	Yes	0	Yes	No	No	No
Canadian Association of Neuropathologists	No	N/A	N/A	Yes	125	Yes	Yes	Yes	No
Canadian Association of Nuclear Medicine	No	N/A	N/A	No	N/A	nd	No	No	No
Canadian Association of Paediatric Surgeons	No	N/A	N/A	Yes	50	Yes	No	Yes	No
Canadian Association of Pathologists	Yes	0	No	Yes	199	Yes	No	No	No
Canadian Association of Physical Medicine & Rehabilitation	Yes	0	Yes	Yes	0	Yes	Yes	Yes	Yes
Canadian Association of Radiation Oncology	No	N/A	N/A	Yes	199	No	Yes	Yes	Yes
Canadian Association of Radiologists	Yes	25	Yes	Yes	150	Yes	No	Yes	Yes
Canadian Association of Thoracic Surgeons	No	N/A	N/A	Yes	350	Yes	No	Yes	nd
Canadian Blood and Marrow Transplant Group	Yes	0	Yes	Yes	180	Yes	No	No	No
Canadian Cardiovascular Society	Yes	0	No	Yes	150	Yes	No	Yes	No
Canadian College of Medical Geneticists	No	N/A	N/A	No	N/A	No	No	No	No
Canadian Critical Care Society	No	N/A	N/A	nd	N/A	No	No	No	No
Canadian Dermatology Association	No	N/A	N/A	Yes	50	Yes	No	No	No
Canadian Fertility and Andrology Society	Yes	25	Yes	Yes	107	Yes	No	No	No
Canadian Geriatrics Society	Yes	0	Yes	Yes	125	Yes	No	Yes	Yes
Canadian Heart Rhythm Society	Yes	0	Yes	Yes	nd	Yes	Yes	Yes	No
Canadian Hematology Society	No	N/A	N/A	nd	nd	nd	nd	nd	No
Canadian Neurological Society	No	N/A	N/A	Yes	nc	nd	No	No	No
Canadian Neurosurgical Society	No	N/A	N/A	Yes	nc	nd	No	No	No
Canadian Ophthalmological Society	No	N/A	N/A	Yes	225	Yes	No	Yes	No
Canadian Orthopaedic Association	No	N/A	N/A	Yes	75	Yes	No	No	No
Canadian Paediatric Society	Yes	65	Yes	Yes	110	Yes	No	Yes	Yes
Canadian Pain Society	Yes	Variable	Yes	Yes	110	Yes	Yes	Yes	No
Canadian Psychiatric Association	Yes	0	Yes	Yes	60	Yes	Yes	Yes	Yes
Canadian Rheumatology Association	No	N/A	N/A	Yes	200	Yes	No	Yes	Yes
Canadian Sleep Society	Yes	40	Yes	Yes	75	Yes	Yes	Yes	No
Canadian Society for Clinical Investigation	No	N/A	N/A	nd	nd	No	No	No	No
Canadian Society for Transfusion Medicine	Yes	60	Yes	Yes	129	Yes	Yes	No	No
Canadian Society for Vascular Surgery	No	N/A	N/A	Yes	nd	Yes	nd	Yes	No
Canadian Society of Addiction Medicine	Yes	0	Yes	Yes	150	Yes	Yes	Yes	No
Canadian Society of Allergy and Clinical Immunology	Yes	0	Yes	Yes	28	Yes	Yes	Yes	No
Canadian Society of Breast Imaging	Yes	0	nd	Yes	0	nd	nd	nd	No
Canadian Society of Cardiac Surgeons	Yes	0	Yes	Yes	150	Yes	Yes	Yes	No
Canadian Society of Clinical Neurophysiologists	No	N/A	N/A	Yes	nc	nd	nd	nd	nd
Canadian Society of Colon and Rectal Surgeons	No	N/A	N/A	Yes	250	Yes	No	Yes	No
Canadian Society of Cytopathology	nd	nd	nd	nd	nc	nd	nd	nd	nd
Canadian Society of Echocardiography	Yes	0	No	Yes	0	Yes	Yes	Yes	No
Canadian Society of Endocrinology & Metabolism	Yes	0	Yes	Yes	120	Yes	No	Yes	No
Canadian Society of Internal Medicine	Yes	0	nd	Yes	nd	Yes	No	Yes	No
Canadian Society of Nephrology	Yes	0	No	Yes	100	N/A	Yes	Yes	No
Canadian Society of Otolaryngology—Head & Neck Surgery	No	N/A	N/A	Yes	25	Yes	No	No	No
Canadian Society of Palliative Care Physicians	Yes	50	Yes	Yes	225	Yes	No	Yes	No
Canadian Society of Pharmacology and Therapeutics	Yes	40	Yes	Yes	75	Yes	Yes	Yes	Yes
Canadian Society of Plastic Surgeons	No	N/A	N/A	Yes	125	Yes	No	Yes	No
Canadian Society of Surgical Oncology	Yes	0	No	Yes	0	nd	nd	nd	No
Canadian Society of Transplantation	Yes	50	nd	Yes	125	Yes	Yes	Yes	No
Canadian Thoracic Society	Yes	59	Yes	Yes	49	Yes	nd	Yes	No
Canadian Urological Association	No	N/A	N/A	Yes	475	nd	No	Yes	Yes
Occupational Medicine Specialists of Canada	Yes	20	No	nd	nd	No	No	No	No
Public Health Physicians of Canada	No	N/A	N/A	No	N/A	N/A	No	No	No
Society of Rural Physicians of Canada	Yes	0	Yes	Yes	nd	Yes	Yes	Yes	Yes
The Canadian Association of Nuclear Medicine	No	N/A	N/A	nd	nd	nd	No	No	No
Society of Gynecologic Oncology of Canada	nd	nd	nd	nd	nd	nd	nd	nd	nd
Society of Obstetricians and Gynaecologists of Canada	Yes	70	Yes	Yes	250	Yes	nd	Yes	Yes
Trauma Association of Canada	Yes	50	Yes	Yes	10	Yes	No	Yes	No
College of Family Physicians of Canada	Yes	0	Yes	Yes	90	Yes	Yes	Yes	Yes

Abbreviations: nd, no data; N/A, not applicable; nc, no conference.

^a^ Conference fee refers to cost of attending virtual conferences in 2020 or 2021.

**Table 2.** Survey of medical organizations for residents.

Society	Membership	Membership Cost (CAD$). Per year	Serve on Committees	Attend Conferences	Conference Fee (CAD$)^a^	Present Research	Travel Award	Research Grant/Award	Mentorship
Association of Medical Microbiology and Infectious Disease Canada	Yes	50	Yes	Yes	100	Yes	Yes	Yes	Yes
Canadian Academy of Child and Adolescent Psychiatry	Yes	0	Yes	Yes	170	Yes	Yes	Yes	Yes
Canadian Academy of Geriatric Psychiatry	Yes	0	Yes	Yes	85	Yes	Yes	Yes	Yes
Canadian Academy of Psychiatry and the Law	Yes	83	nd	Yes	125	Yes	Yes	Yes	No
Canadian Academy of Sport and Exercise Medicine	Yes	175	nd	Yes	200	Yes	No	No	No
Canadian Anesthesiologists’ Society	Yes	0	Yes	Yes	40	Yes	nd	Yes	Yes
Canadian Association for the Study of Liver	Yes	0	Yes	Yes	0	Yes	Yes	Yes	No
Canadian Association of Child Neurology	Yes	80	Yes	Yes	nc	Yes	Yes	Yes	Yes
Canadian Association of Emergency Physicians	Yes	140	Yes	Yes	288	Yes	No	Yes	Yes
Canadian Association of Gastroenterology	Yes	50	Yes	Yes	100	Yes	Yes	Yes	Yes
Canadian Association of General Surgeons	Yes	0	Yes	Yes	250	Yes	Yes	Yes	Yes
Canadian Association of Interventional Cardiology	Yes	nd	nd	nd	nd	nd	nd	nd	nd
Canadian Association of Interventional Radiology	Yes	57	Yes	Yes	0	Yes	Yes	nd	No
Canadian Association of Medical Biochemists	nd	nd	nd	Yes	nd	Yes	nd	nd	nd
Canadian Association of Medical Oncologists	Yes	0	Yes	Yes	0	Yes	No	Yes	Yes
Canadian Association of Neuropathologists	Yes	0	No	Yes	125	Yes	Yes	Yes	No
Canadian Association of Nuclear Medicine	Yes	0	No	Yes	0	Yes	No	Yes	No
Canadian Association of Paediatric Surgeons	No	N/A	N/A	Yes	50	Yes	No	Yes	No
Canadian Association of Pathologists	Yes	38	Yes	Yes	199	Yes	Yes	Yes	No
Canadian Association of Physical Medicine & Rehabilitation	Yes	20	Yes	Yes	0	Yes	Yes	Yes	Yes
Canadian Association of Radiation Oncology	Yes	0	Yes	Yes	350	Yes	Yes	Yes	No
Canadian Association of Radiologists	Yes	25	No	Yes	150	Yes	No	Yes	Yes
Canadian Association of Thoracic Surgeons	Yes	0	No	Yes	350	Yes	Yes	Yes	Yes
Cell Therapy Transplant Canada	Yes	0	Yes	Yes	180	Yes	No	No	No
Canadian Cardiovascular Society	Yes	0	Yes	Yes	240	Yes	Yes	Yes	Yes
Canadian College of Medical Geneticists	Yes	0	Yes	Yes	150	Yes	No	Yes	No
Canadian Critical Care Society	Yes	50	nd	Yes	465	Yes	No	No	No
Canadian Dermatology Association	Yes	0	No	Yes	50	Yes	No	Yes	No
Canadian Fertility and Andrology Society	Yes	125	Yes	Yes	160	Yes	No	Yes	No
Canadian Geriatrics Society	Yes	50	Yes	Yes	140	Yes	No	Yes	Yes
Canadian Heart Rhythm Society	Yes	0	Yes	Yes	nd	Yes	Yes	Yes	No
Canadian Hematology Society	Yes	0	Yes	nd	nd	nd	nd	nd	No
Canadian Neurological Society	Yes	80	Yes	Yes	nc	Yes	Yes	Yes	No
Canadian Neurosurgical Society	Yes	80	Yes	Yes	nc	Yes	Yes	Yes	No
Canadian Ophthalmological Society	Yes	0	Yes	Yes	225	Yes	No	Yes	No
Canadian Orthopaedic Association	Yes	0	No	Yes	150	Yes	No	No	Yes
Canadian Paediatric Society	Yes	65	Yes	Yes	199	Yes	Yes	Yes	Yes
Canadian Pain Society	Yes	Variable	Yes	Yes	110	Yes	Yes	Yes	No
Canadian Psychiatric Association	Yes	60	Yes	Yes	60	Yes	Yes	Yes	Yes
Canadian Rheumatology Association	Yes	0	Yes	Yes	200	Yes	Yes	Yes	Yes
Canadian Sleep Society	Yes	40	Yes	Yes	75	Yes	Yes	Yes	No
Canadian Society for Clinical Investigation	Yes	50	nd	Yes	nd	Yes	No	Yes	No
Canadian Society for Transfusion Medicine	Yes	60	Yes	Yes	129	Yes	Yes	No	No
Canadian Society for Vascular Surgery	Yes	0	nd	Yes	nd	Yes	nd	Yes	No
Canadian Society of Addiction Medicine	Yes	45	Yes	Yes	250	Yes	Yes	Yes	No
Canadian Society of Allergy and Clinical Immunology	Yes	0	Yes	Yes	28	Yes	Yes	Yes	No
Canadian Society of Breast Imaging	Yes	0	nd	Yes	0	nd	nd	nd	No
Canadian Society of Cardiac Surgeons	Yes	0	Yes	Yes	240	Yes	Yes	Yes	Yes
Canadian Society of Clinical Neurophysiologists	No	N/A	N/A	Yes	nc	nd	nd	nd	nd
Canadian Society of Colon and Rectal Surgeons	Yes	0	Yes	Yes	250	Yes	No	Yes	No
Canadian Society of Cytopathology	Yes	0	Yes	nd	nc	nd	nd	nd	nd
Canadian Society of Echocardiography	Yes	0	Yes	Yes	0	Yes	Yes	Yes	No
Canadian Society of Endocrinology & Metabolism	Yes	0	Yes	Yes	120	Yes	Yes	Yes	No
Canadian Society of Internal Medicine	Yes	0	Yes	Yes	nd	Yes	No	Yes	No
Canadian Society of Nephrology	Yes	0	No	Yes	100	N/A	Yes	Yes	No
Canadian Society of Otolaryngology—Head & Neck Surgery	Yes	0	Yes	Yes	25	Yes	Yes	Yes	No
Canadian Society of Palliative Care Physicians	Yes	50	Yes	Yes	225	Yes	Yes	Yes	No
Canadian Society of Pharmacology and Therapeutics	Yes	75	Yes	Yes	75	Yes	Yes	Yes	Yes
Canadian Society of Plastic Surgeons	Yes	0	Yes	Yes	125	Yes	No	Yes	No
Canadian Society of Surgical Oncology	Yes	0	No	Yes	0	nd	nd	nd	Yes
Canadian Society of Transplantation	Yes	50	nd	Yes	125	Yes	Yes	Yes	No
Canadian Thoracic Society	Yes	99	Yes	Yes	49	Yes	nd	Yes	No
Canadian Urological Association	Yes	0	Yes	Yes	475	nd	No	Yes	Yes
Occupational Medicine Specialists of Canada	Yes	50	No	nd	nd	No	No	No	No
Public Health Physicians of Canada	Yes	0	Yes	Yes	75	nd	Yes	Yes	Yes
Society of Rural Physicians of Canada	Yes	20	Yes	Yes	nd	Yes	Yes	Yes	Yes
The Canadian Association of Nuclear Medicine	Yes	0	Yes	Yes	nd	Yes	No	Yes	No
Society of Gynecologic Oncology of Canada	nd	nd	nd	nd	nd	nd	nd	nd	nd
Society of Obstetricians and Gynaecologists of Canada	Yes	85	Yes	Yes	250	Yes	nd	Yes	No
Trauma Association of Canada	Yes	50	Yes	Yes	150	Yes	No	Yes	No
College of Family Physicians of Canada	Yes	102	Yes	Yes	240	Yes	Yes	Yes	Yes

Abbreviations: nd, no data; N/A, not applicable; nc, no conference.

^a^ Conference fee refers to cost of attending virtual conferences in 2020 or 2021.

Most organizations allow trainees to attend their annual conference (83% for students and 93% for residents), and the mean (SD) cost of attending the most recent virtual conference was $114 ($98) for students, ranging from $0 to $475, and $142 ($113) for residents, ranging from $0 to $475. Free conference attendance is offered in 8 (11%) organizations for both medical students and residents. Medical students can present research at the annual conferences of 48 (68%) organizations and residents can present research at 60 (85%). Research funding or awards are available in 41 (58%) organizations for medical students and 56 (79%) for residents. Travel awards to in-person conferences are available in 22 (31%) organizations for medical students and 37 (52%) for residents.

## Discussion

This study evaluated the scholarly enrichment opportunities offered by Canadian medical specialty organizations to medical students and residents. We found that both medical students and residents have numerous opportunities to become trainee members of medical organizations, attend conferences, present research, and apply for travel and research awards. When comparing the two types of trainees, opportunities were more often available to residents than they were for medical students.

Based on current medical education literature, it is unclear whether involvement in medical specialty organizations is associated with increased trainee competency or interest in the specialty. However, it has been demonstrated that formal mentorship programs are associated with increased career satisfaction and superior career options for trainees.^7^ In our study, several organizations had mentorship programs to guide medical students, residents, or early career attending physicians in their desired specialty. Additionally, opportunities to present research at academic conferences may enhance medical student applications for the residency selection process.^8^ By presenting research in their desired specialty, students can demonstrate an interest in the field and network with physicians in that organization. Furthermore, involvement in academic conferences may increase the likelihood of a trainee pursuing a career in academic medicine,^8^ something that may be seen as desirable for residency programs.

The present study has several limitations that should be considered when evaluating our results. First, our study is limited by the scope of its investigation. With our aim to provide a general overview of what is available for medical students and residents in Canada, only organizations at the national level were assessed. Trainees should also explore opportunities to get involved in their specialty of choice at the regional, provincial, or territorial level. Furthermore, our study is limited by our method of data collection, which used the organizations’ websites that may not perfectly reflect the opportunities available. Furthermore, although several attempts were made to contact each organization, some were unable to verify the data that had been collected. In cases where reviewers did not agree on the data point or it remained unclear, it was described in our results as no data.

## Conclusion

Educational and leadership opportunities are important for the professional development of trainee physicians. Although many scholarly enrichment opportunities already exist for medical students and residents, there appears to be room for expansion within Canadian organizations. The involvement of trainees in medical specialty organizations should continue to be encouraged to foster the growth of medical students and resident physicians.

## References

[1] Foulad DP Fouladian JE Hakimi AA Mesinkovska NA. Educational and scholarly opportunities for medical students and residents in the United States dermatology societies. J Am Acad Dermatol. 2022;86(4):894–897. 10.1016/j.jaad.2021.03.027. 33722551

[2] Dy CJ Cross MB Osbahr DC Parks ML Green DW. What opportunities are available for resident involvement in national orthopedic and subspecialty societies? Orthopedics. 2011;34(10):e669–e673. 10.3928/01477447-20110826-13. 21956064

[3] Wong K Jang M Gilad A Levi JR. Resident involvement in professional otolaryngology organizations: current trends in the United States. Otolaryngol—Head Neck Surg. 2017;157(2):222–225. 10.1177/0194599817703077. 28417658

[4] Yang A Zhang X Liu J Chow J Adelman R Forster S. Educational and scholarly opportunities for medical students and residents in professional ophthalmology organizations. JAMA Ophthalmol. 2018;136(12):1417–1419. 10.1001/jamaophthalmol.2018.3963. 30326048 PMC6594420

[5] Yasmeh JP Bakhshizadeh O Azhdam AM Hakimi AA. Scholarly opportunities for medical students and residents in United States professional radiology organizations. Clin Imaging. 2021;80:199–201. 10.1016/j.clinimag.2021.07.018. 34340202

[6] National Specialty Societies. The Royal College of Physicians and Surgeons of Canada. Accessed July 24, 2021. https://www.royalcollege.ca/rcsite/resources/national-specialty-societies-e

[7] Bredella MA Fessell D Thrall JH. Mentorship in academic radiology: why it matters. Insights Imaging. 2019;10(1):1–7. 10.1186/s13244-019-0799-2. 31728762 PMC6856244

[8] Grubbs JR Mian SI. Advising students interested in ophthalmology: a summary of the evidence. Ophthalmology. 2016;123(7):1406–1410. 10.1016/j.ophtha.2016.04.016. 27342323

